# Kimura's Disease: A Literature Review Based on a Clinical Case

**DOI:** 10.7759/cureus.50463

**Published:** 2023-12-13

**Authors:** Maria Teresa Brito, Diana Baptista, Edite Pereira, Elsa Fonseca, Jorge S Almeida

**Affiliations:** 1 Internal Medicine, Centro Hospitalar Univeristário de São João, Porto, PRT; 2 Pathologic Anatomy, Centro Hospitalar Univeristário de São João, Porto, PRT; 3 Medicine, Faculdade de Medicina da Universidade do Porto (FMUP), Porto, PRT

**Keywords:** ige, lymph node, subcutaneous swelling, hypereosinophilia, kimura disease

## Abstract

Kimura's disease (KD) was first described in 1937. It is a rare, benign, and chronic immune-mediated inflammatory disorder affecting the subcutaneous tissue, salivary glands, and lymph nodes. The disease is more common in the second to third decades of life in middle-aged Southeast Asian countries. The cause of Kimura's disease remains unknown; some authors believe it is related to an autoimmune or delayed-type hypersensitivity reaction. It commonly presents as a solitary painless lymph node in the head and neck or generalized lymphadenopathy (67%-100%) associated with peripheral eosinophilia and elevated IgE levels. Renal involvement may occur in some patients. Diagnosis is made by histology.

A 21-year-old Caucasian man with no relevant medical history presented with a non-tender swelling of the left hemiface without other associated symptoms. Laboratory investigations revealed a leukocyte count with eosinophilia (2.29×10^9/L- 26.5%) and elevated total IgE and IgG4. He had no renal dysfunction. He underwent surgical resection of the lesion in the jugal, infraorbital, and left nasolabial regions, and the anatomopathological examination revealed the characteristics of Kimura's disease. Three months after surgery, an MRI showed an increase in the size of the mass, and he was started on corticosteroids. Six months after surgery, he presented with a slight increase in the size of the mass and was started on Ciclosporin, which allowed a progressive reduction in the dose of corticosteroid with evidence of a progressive reduction in swelling. Ciclosporin was stopped due to toxic serum levels, and he was started on mycophenolate mofetil. The dose was increased because of the increase in facial mass; on mycophenolate mofetil 2500mg/day, the patient remains stable.

KD is a chronic disorder of unknown etiology that mainly affects young people in Southeast Asia and is rare in Western countries, as in the case of this young man. Studies have shown no significant differences in region or race, complications, multiplicity, laterality, anatomical distribution, maximum size, eosinophil count, or IgE levels between age groups. There is no consensus on the optimal treatment for KD; several treatments have been used, including surgery, systemic corticosteroids, immunosuppressants, and radiation. Due to the tendency to relapse and the rarity of the disease, there is no consensus on treatment options for relapse.

## Introduction

Kimura's disease (KD) was first described in the Chinese literature in 1937 by Kim and Szeto as "hyperplastic lymphogranuloma with eosinophilia" [1].

It is a rare, benign, chronic, immune-mediated inflammatory disorder affecting the subcutaneous tissue, salivary glands, and lymph nodes [2]. It is a benign idiopathic chronic inflammatory disorder with fewer than 200 reported cases worldwide that may mimic a neoplastic condition [3].

The disease usually affects young men in Southeast Asian countries (but rarely in Indians) and is rare in Western countries [1,3]. Most patients are in their second to third decade of life, with a male preponderance (male to female ratio: 3.5-7:1) [3].

The cause of Kimura's disease is still unknown [2]. Infectious etiologies have been postulated, but it is now thought that KD is related to an autoimmune or delayed-type hypersensitivity reaction. The association with asthma, allergic rhinitis and conjunctivitis, atopic dermatitis, peripheral hypereosinophilia, and elevated serum IgE levels supports the idea of an aberrant allergic response [4]. Some have suggested a role for interleukins (IL-4 and IL-5) and mast cells in regulating immunoglobulin (IgE) synthesis and eosinophil infiltration [2]. Other causes, such as Candida infection, arthropod bites, vírus, neoplasms, deregulation of eosinophil dynamics and IgE synthesis, and altered systemic immune-mediated response, have all been postulated as causative [5].

The reactive nature of this disease is reflected in the distinct histopathological patterns of florid follicular hyperplasia, inter- and intrafollicular dense eosinophilic and lymphoplasmacytic infiltration, eosinophilic microabscesses (with or without Charcot-Leyden crystals), increased small blood vessels, and prominent stromal fibrosis. Lymphoproliferation is associated with the proliferation of post-capillary venules. However, high endothelial venules are atypical. Replacement of normal germinal centers with IgE or eosinophilic deposition, necrosis, and polykaryocytes may be present. In some patients, inflammatory infiltration of nerve fibers leads to skin irritation and pruritus [6].

Most patients do not have systemic symptoms [6]. The typical presentation is a solitary enlarged painless lymph node or generalized lymphadenopathy (67% to 100%) presenting as non-tender subcutaneous swelling of the head and neck, predominantly in the periauricular, submandibular, inguinal, orbital, and eyelid areas. It may be associated with or without salivary gland involvement, marked peripheral eosinophilia, and elevated IgE levels [1]. In non-Asian patients, the salivary glands are rarely affected, although the lacrimal glands are involved in some cases [7].

Visceral involvement is a rare complication and may occur late in the course of the disease, warranting long-term follow-up [6]. Renal disease is common (incidence of 10% to 60%), ranging from urinalysis abnormalities to renal insufficiency (reported in rare cases) [6]. In some cases, albuminuria has preceded skin lesions [7]. Kidney damage is probably due to immune complex-mediated damage or Th2-dominated immune response disorders [5]. Several pathologies have been associated with KD, including focal and segmental glomerulosclerosis, membranous glomerulonephritis, membranoproliferative glomerulonephritis, eosinophilic tubulointerstitial nephritis, minimal change disease, and acute tubular necrosis [6].

Other systemic manifestations have been reported, including asthma, tuberculosis, Loeffler's syndrome, and even allergy to fungal infections such as *Candida* [4].

*Peripheral eosinophilia* and *Eosinophils* in the inflammatory infiltrate suggest that KD may be a type of hypersensitivity reaction [5]. Ultrasound, computed tomography (CT), and magnetic resonance imaging (MRI) can be diagnostic and help to stage the extent and progression of the disease and lymph node involvement [5]. On radiological examination, Kimura's disease may be confused with other malignancies, such as lymphoma [8].

The definitive diagnosis can be confirmed histologically by biopsy or excision of the involved mass [9]. The histological features of Kimura's disease are classified as constant (including preserved nodal architecture, florid germinal center hyperplasia, eosinophilic infiltration, and postcapillary venous proliferation), frequent (sclerosis, polykaryocytes, germinal center vascularization, postcapillary venous proliferation), germinal center vascularization, proteinaceous deposits in germinal centers, germinal center necrosis, eosinophilic abscesses, and reticular IgE deposits in germinal centers), and rare (progressive transformation of germinal centers). Nodal architecture is largely preserved in most cases, but capsular fibrosis with subcapsular sinusoidal obliteration and perinodal soft tissue involvement is common [1].

The differential diagnosis of this condition should include angiofollicular hyperplasia with eosinophilia (ALHE), eosinophilic granuloma, benign lymphoepithelial lesion, lymphocytoma, pyogenic granuloma, Hodgkin's disease, Kaposi's sarcoma, malignant tumors of cutaneous or subcutaneous origin, metastatic tumors, harmartoma, epithelioid haemangioma, Castleman's disease, tuberculosis, dermatopathic lymphadenopathy, Langerhan's cell histiocytosis, parasitic lymphadenitis, drug-induced lymphadenopathy, parasitic lymphadenitis, and lymphoma [10].

The closest differential diagnosis is ALHE; both conditions present as soft tissue swelling of the head and neck with a prolonged indolent clinical course, and both show eosinophilic infiltrates and vascular proliferation. However, KD occurs predominantly in Asians with a male predilection, peripheral eosinophilia, and elevated serum IgE levels [1]. The solitary lesions are usually located in the subcutaneous tissue and are often associated with regional lymphadenopathy and salivary gland involvement. ALHE occurs in all racial groups with a slight female predominance; patients present with small superficial dermal papulonodules accompanied by bleeding, pruritus, and tumor growth. Regional lymphadenopathy, serum eosinophilia, and elevated IgE levels are rare. Histologically, KD has three components: cellular (inflammatory infiltrate with increased eosinophils and follicular hyperplasia), fibrocollagenous, and vascular (arborizing vascular proliferation of the postcapillary venule; endothelial cells are usually flat and lack cytological atypia or vacuolization), in contrast to the vascular proliferation seen in ALHE [1].

Some features of KD are of diagnostic importance and may help to distinguish it from ALHE, such as the presence of abundant fibrous tissue and lymphoid follicles, the tissue in lesions of both very short and long duration, tissue eosinophilia, which is always present and may include eosinophilic abscesses, and capillary proliferation with large, thick-walled vessels [11].

There is currently no definitive treatment for KD [10]. Treatment of the disease depends on the clinical presentation and is not uniform. Surgical excision is often the first-line treatment, especially for small and accessible lesions, with a 100% success rate but a 42%-66% recurrence rate [12]. Complete surgical clearance is challenging due to the infiltrative nature of the disease and regional lymphadenopathy [3].

Treatment with oral prednisolone, trans-retinoic acid, leukotriene receptor antagonists such as montelukast, thalidomide, cyclosporine, interferon-alpha, omalizumab (an anti-IgE antibody), and H1 receptor blockers may be tried [4]. Local radiotherapy may be considered first-line adjuvant therapy after surgery or as a stand-alone modality for recurrent or refractory disease [3].

The clinical course is generally benign and self-limited. It is chronic, characterized by episodes of remission or even spontaneous regression and recurrence [3], and no cases of malignant transformation have been reported [11]. Most patients have a gradual increase in swelling, but spontaneous resolution is occasionally seen [1]. Recurrence rates can be as high as 60%, even after complete excision of the lesion [2]. Eosinophil count is considered a useful predictive marker [7].

## Case presentation

A 21-year-old man of Caucasian man with no relevant medical history presented with a non-tender swelling of the left hemiface without other associated symptoms.

Laboratory examination showed a serum white blood cell count of 8.64×10^9/L, with eosinophilia (2.29×10^9/L, or 26.5%). Total serum IgE was elevated at 6980 kU/L (maximum 114), and IgG4 was elevated at 267 kU/L (maximum 140). Erythrocyte sedimentation rate, liver and thyroid function tests, creatine phosphokinase, and lactate dehydrogenase were normal. Renal function was normal, with no proteinuria in 24-hour urine, negative anti-neutrophil cytoplasmic antibodies, and no relevant morpho-functional changes on renovesical ultrasound.

**Figure 1:**
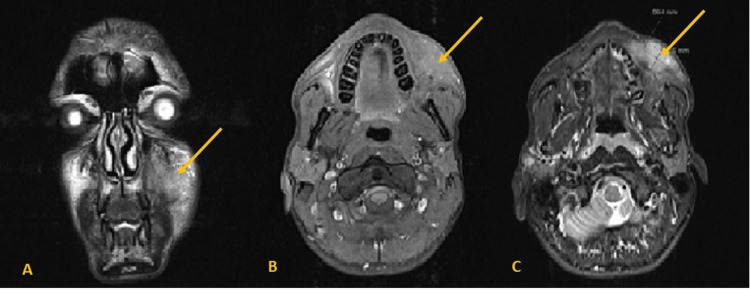
Face MRI Initial MRI of the face A: T2 weighting with fat saturation, B: T1 weighting after contrast, C: T2 weighting

He underwent a facial MRI, which showed a mass in the left hemiface located in the space masticator, anterior to the masseter, between the skin and the jaw, and elevation of the buccal angle. This mass has an irregular configuration and measures 56mm (AP) x 20mm (T) x 55mm (cc). In the upper part, a notch is outlined up to the midline and the base of the orbit, which it does not reach, in a topography that seems to correspond to the second branch of the trigeminal nerve; in the deep interface, it is possible to note the blurring of the plane muscular tissue, apparently due to invasion, leaving the plan intact as regards the signal characteristics: a hyposignal on T1 and an intermediate signal or slight hypersignal on T2-weighted sequences. The neoformation has a global and homogeneous shape and contrast (Figure 1).

**Figure 2:**
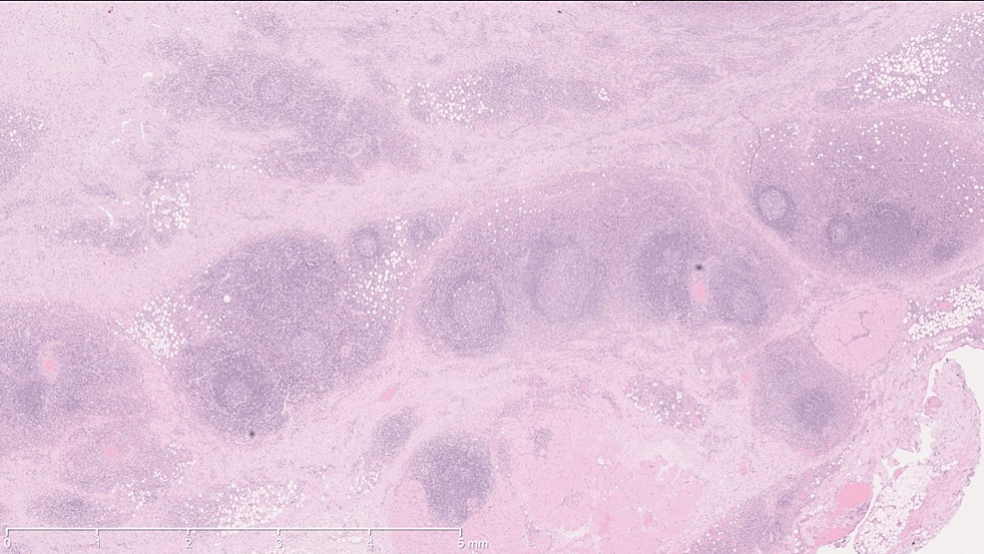
Nodular growth Nodular growth affects deeper areas (muscles) and is separated by fibrous bundles. The stain used was hematoxylin-eosin. The scale used in mm is shown in the figure.

**Figure 3:**
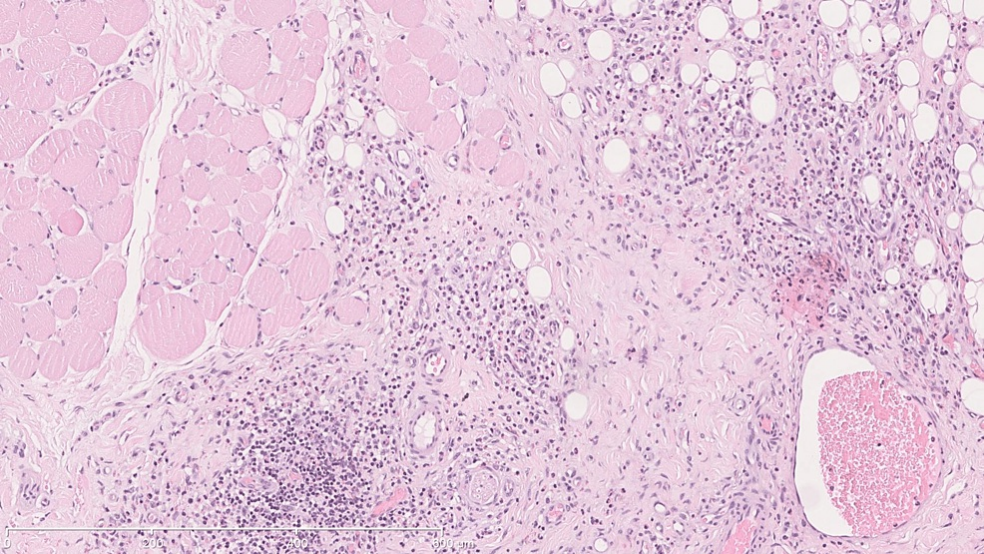
Infiltrate and muscle The stain used was hematoxylin-eosin. The scale used in mm is shown in the figure.

**Figure 4:**
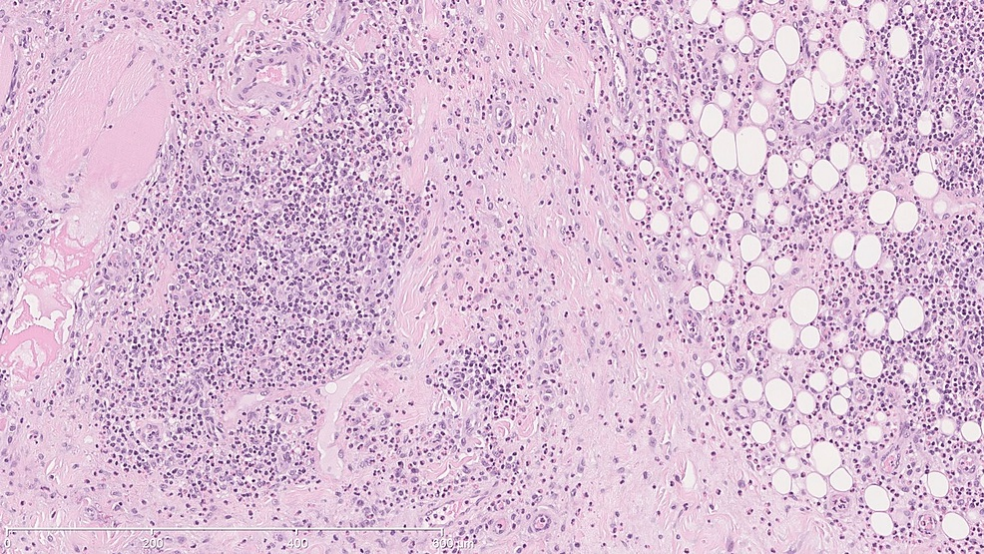
Eosinophilic infiltrate in adipose tissue The stain used was hematoxylin-eosin. The scale used in mm is shown in the figure.

**Figure 5:**
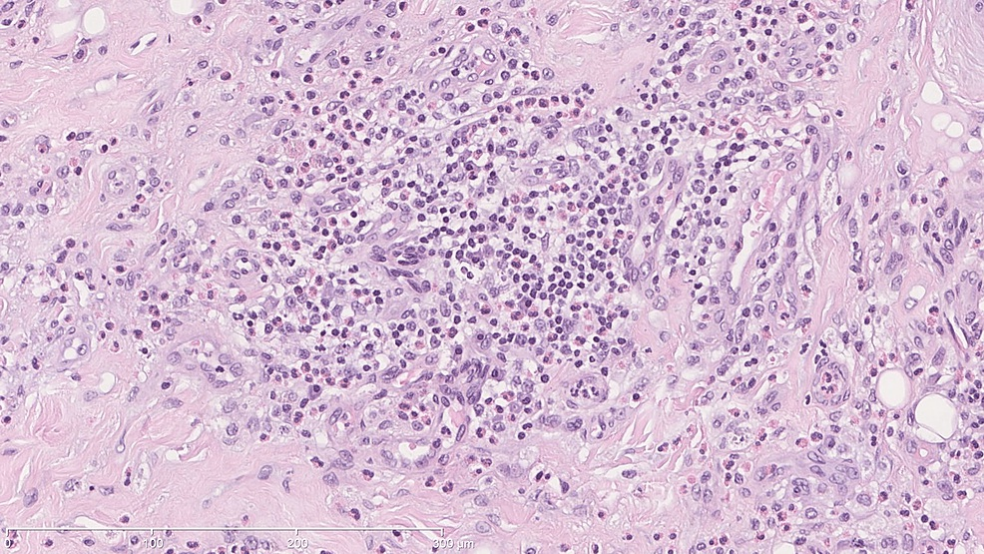
Eosinophilic microabscesses The stain used was hematoxylin-eosin. The scale used in mm is shown in the figure.

He underwent surgical resection of the lesion in the jugal, infraorbital, and left nasolabial regions. Anatomopathological examination revealed lymphoid hyperplasia with eosinophilia involving the left cheek. The features observed suggest Kimura's disease as the most likely hypothesis, or alternatively, angiolymphoid hyperplasia with eosinophilia/epithelial hemangioma. There was no evidence of a lymphoproliferative process (Figures 2-5).

The clinical and histological data and the elevated IgE concentration allowed us to confirm a case of Kimura's disease.

**Figure 6:**
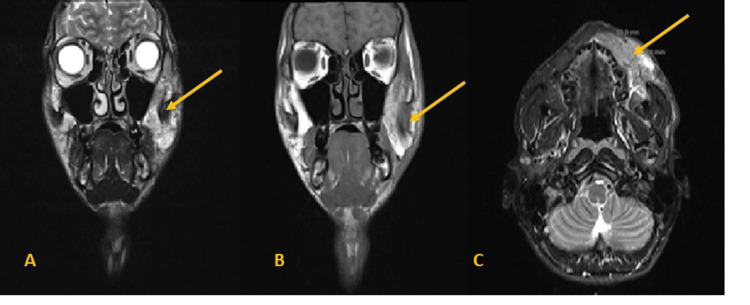
MRI of the face A: T2 weighting with fat saturation, B: T1 weighting after contrast, C: T2 weighting

Three months after the operation, the facial MRI was repeated: "Compared with the previous examination, there was an increase in the dimensions of the mass on the left hemiface, located in the masticatory space, anterior to the masseter, and between the skin and the maxillary and mandibular/buccinator muscles, and an elevation of the buccal angle. This mass measures 60x25x65cm (measured on the same axes at 56x20x55mm). In the upper part, it touches the midline and the base of the orbit, which it does not reach" (Figure 6).

He was then treated with corticosteroids (prednisolone, maximum dose 30mg/day, a dose that was maintained for six months) with improvement of the swelling. With the start of corticosteroid therapy, there was a resolution of the hypereosinophilia levels, which remained low even after the dose was reduced. No adverse effects of corticosteroid therapy were reported.

Six months after surgery, a facial MRI showed a slight increase in the size of the mass, and treatment with cyclosporine was started. The initial dose was 100mg/day, later increased to 200mg/day, allowing a progressive reduction in the dose of corticosteroid therapy and evidence of a progressive reduction in swelling.

Due to toxic levels of the drug, he reduced the cyclosporine dose to 100mg/day, resulting in a progressive increase in mass. Cyclosporine was stopped, and mycophenolate mofetil (MMF) was started (progressively increased over five months to a maximum dose of 2000mg/day).

Seven months after starting MMF, he had a new mass increase, and the dose of MMF was increased to 2500mg/day. He underwent a repeat facial MRI three months later, which showed no increase in lesion size. The patient remains under observation.

## Discussion

KD is a chronic inflammatory disease of unknown etiology that presents with tumor-like swellings [1]. Despite its rarity, it mainly affects young people in Southeast Asia and is rare in Western countries, as in the case of this young man. Zhang et al. published the longest case series (46 cases) and found that KD is more common in males in the second and third decades of life; the most common sites were the head and neck (63%), and only 13% of patients had inguinal adenopathies [13].

The 2019 study by Kakehi et al. found age-related differences in sex ratio, frequency of pruritus, and time to diagnosis in patients with KD. No significant differences were found in region, race, complications, multiplicity, laterality, anatomical distribution, size, eosinophil count, or IgE levels between age groups. The male:female ratio appears to decrease with age. Sex hormone receptors are expressed on mast cells, but whether this is associated with the age-related change in sex ratio in patients with KD remains unknown [8].

Our patient had all the typical features of KD and no systemic manifestations. Kidney failure may be observed at the same time as or long after the appearance of the masses, and proteinuria is seen in 12%-16% of patients, of whom 60%-80% have nephrotic syndrome [5]. Membranous glomerulonephritis is the most frequent histopathological pattern [13]. In our patient, a renal examination was performed by Doppler ultrasound, but no renal abnormalities were found. In addition, his serum creatinine and urine protein concentrations were within reference ranges.

KD usually presents as a painless mass or subcutaneous nodule in the head and neck (in up to 76% of patients), with occasional pruritus of the overlying skin; the study by Kakehi et al. showed that pruritus increased with age [8]. The fact that pruritus often occurs in older patients due to humoral and cellular immune defects with eosinophilia and hyper-IgE may be partly related to the effects of age on pruritus in KD [8].

IgG4-related disease features may be important confounders in KD lesions. In IgG4-related disease, the vast majority of cases had enlargement of multiple salivary and/or lacrimal glands, and most patients had bilateral submandibular gland involvement. In KD, unilateral parotid involvement and associated skin lesions were present in more than half of the patients. In IgG4-related disease, serum IgG4 levels were elevated in >90% of cases compared to only 19% in KD [14]; in our case, the patient had high IgG4 levels.

Elevated eosinophil counts and IgE concentrations were found in most patients with KD (86% and 96%, respectively), but also, although less frequently, in IgG4-related disease (23% and 77%, respectively). In the latter, storiform fibrosis, irregular lymphoid follicles, and increased IgG4-positive cells were common, whereas in KD, acellular fibrosis, regular lymphoid follicles, IgE-positive reticular networks, increased IgE-positive cells, and tryptase-positive mast cells were more common [14]. Serum IgE was associated with mass size and therapeutic response in KD, but eosinophil count may be considered a useful predictive marker [7].

Due to the lack of large-scale systemic clinical trials, there is no consensus on the optimal treatment for KD [15]. There have been many treatment options, including surgical excision, systemic corticosteroids, immunosuppressants (cyclosporine, azathioprine, and cyclophosphamide), and radiation [8]. Due to the tendency to recur and the rarity of the disease, there is no consensus on treatment options for recurrence [3].

Surgery may be considered first, especially for localized lesions, even if recurrence is possible (42%-66%), and is the most commonly used treatment, helping to achieve a definitive diagnosis, although recurrence is common [12]. In patients with a maximum tumor diameter of <3 cm, surgery is an effective single treatment [16]. However, in patients with larger and infiltrative lesions, complete surgical removal is limited, and recurrence can occur rapidly. Large-volume resections may contribute to cosmetic and functional disability [16].

Patients with risk factors for recurrence, especially those with positive margins, may require postoperative adjuvant treatment, such as radiotherapy and systemic immunosuppressive drugs [16].

Corticosteroid therapy has been used particularly in patients with multiple organ involvement, but relapses often occur when the dose is reduced or stopped [16]. Corticosteroid therapy alone has not shown convincing results due to its transient effects [17]. Intralesional injections of corticosteroids have also shown good results [17]. Nakahara et al. reported that steroid therapy can control lesions, lymphadenopathy, and nephrotic syndrome, but local recurrence was common during the period of steroid tapering [18].

Radiotherapy provides local control and is used in patients with incomplete resection or recurrence, steroid-resistant lesions, and/or inaccessible or too extensive lesions for surgical treatment [14]. It has not been the most widely used option due to its cosmetic outcome and toxicities, including the risk of second malignancies [3]. The observed recurrence rate is 30% to 50% [12]. A recent study showed that radiotherapy (20-45 Gy) was more effective than local excision and steroid treatment, with a local response rate of 64.3% (versus 22.2% in the steroid group) [7]. No adverse events were reported in the radiotherapy group during follow-up (65 months) [7].

The 2020 study by Zhang et al. showed that the recurrence rate after surgical excision combined with low-dose radiotherapy was lower than that of surgical excision alone or surgical excision followed by oral corticosteroids [15].

In a case series with 17 patients with recurrent KD, cases treated with steroids alone had rapid recurrence, with a worse failure rate in the non-irradiated group (75%) than in the irradiated group (11%) [3]. In another study, similar results were reported with complete remission in 64% of patients in the radiotherapy group compared with 22% in the non-radiotherapy group [3].

Other systemic immunosuppressive drugs such as cyclosporine, mycophenolate mofetil, mycophenolic acid, leflunomide, and tacrolimus have also been reported to be effective in some cases [16]. Retinoids, monoclonal antibodies (imatinib), antihistamines (cetirizine), pentoxifylline, photodynamic therapy, and cryotherapy have also been used [8]. Patients successfully treated with omalizumab, benralizumab, dupilumab, and mepolizumab have also been reported in the literature [19]. Cyclosporine may be an effective drug for KD because of its effect on Th2 cells; some patients have been reported to be cured with cyclosporine (5 mg/kg per day) [7].

Sun et al. reported that imatinib may be an effective drug for the treatment of the disease [7]. Some biologics, including anti-IgE, anti-IL-5, and anti-IL-4/IL-13, have been used to treat KD [19]. However, the results of trials in these patients treated with biologics are still controversial [14,19].

Dupilumab, a monoclonal antibody that blocks IL-4 and IL-13, was administered at an initial dose of 600 mg followed by 300 mg every two weeks for four months and showed effects with reduced masses and a rapid decrease in eosinophil counts, while serum IgE levels remained high [14]. Mepolizumab appears to reduce the size and density of the mass, with the pathological changes being reduced eosinophils in the tissue rather than fibrosis. Benralizumab also appears to have a long-term effect on clinical improvement [19].

A combination of surgery and radiotherapy appears to be the best treatment for KD [15], with observation often sufficient in asymptomatic cases [17].

In a meta-analysis of 31 studies by Lee et al., the recurrence rate of surgical excision was 30.5%, medical therapy was 45%, and the combination of surgery and postoperative adjuvant therapy had the lowest recurrence rate of 26.94% [20].

Recurrence occurs in up to 25% of patients with KD and is influenced by some factors such as disease duration, blood eosinophil count, IgE level, definition of lesion boundaries, single versus multiple lesions, and lesion diameter [8]. Outcomes are also influenced by other factors such as other systemic diseases, lifestyle (e.g., smoking), surgery alone, and type of treatment [8].

Despite being a benign disease, the recurrence of KD is very common (up to 60%-80%) [13]. The course of the disease is chronic, with periods of remission or even spontaneous regression and recurrence [12]. The overall outcome is good as it is not associated with malignancy [17].

In this patient, surgery was very important to confirm the diagnosis and was the first line of therapy. Due to a relapse three months after surgery, it was necessary to start systemic treatment, first with corticosteroids and then with another immunosuppressant. The best treatment for this patient was initially challenging, but the patient is currently stable and under observation.

## Conclusions

Kimura's disease is rare, even more so in non-Asian patients. The diagnosis can only be confirmed by histopathological examination. There are several treatment options, but there is still no consensus on the best treatment, which must be appropriate for each patient and the clinical context.
